# Endoplasmic reticulum stress and the unfolded protein response in skeletal muscle of subjects suffering from peritoneal sepsis

**DOI:** 10.1038/s41598-021-04517-9

**Published:** 2022-01-11

**Authors:** Uta Barbara Metzing, Christian von Loeffelholz, Ricardo Steidl, Bernd Romeike, René Winkler, Falk Rauchfuß, Utz Settmacher, Christian Stoppe, Sina M. Coldewey, Claudia Weinmann, Martin O. Weickert, Ralf A. Claus, Andreas L. Birkenfeld, Christian Kosan, Paul Horn

**Affiliations:** 1 Department of Trauma, Hand and Reconstructive Surgery, Jena University Hospital, Friedrich Schiller University, Jena, Germany; 2 Department of Anesthesiology and Intensive Care, Jena University Hospital, Friedrich Schiller University, Am Klinikum 1, 07747 Jena, Germany; 3 Department of Anaesthesiology, Intensive Care, Pain Medicine and Emergency Medicine, Bundeswehrkrankenhaus Berlin, Berlin, Germany; 4 Section of Neuropathology, Department of Pathology, Jena University Hospital, Jena, Germany; 5 Dean’s Office, Medical Didactics, University Rostock Medical Center, Rostock, Germany; 6 Department of Biochemistry, Center for Molecular Biomedicine (CMB), Friedrich-Schiller-University Jena, Jena, Germany; 7 Department of General, Visceral and Vascular Surgery, Jena University Hospital, Jena, Germany; 8 Department of Anesthesiology and Intensive Care Medicine Wuerzburg, University Hospital, Wuerzburg, Germany; 9 3CARE-Cardiovascular Critical Care & Anesthesia Evaluation and Research, Medical Faculty RWTH Aachen, Aachen, Germany; 10 Septomics Research Centre, Jena University Hospital, Jena, Germany; 11 Center for Sepsis Control and Care, Jena University Hospital, Jena, Germany; 12 Warwickshire Institute for the Study of Diabetes, Endocrinology and Metabolism, University Hospitals Coventry and Warwickshire NHS Trust, Coventry, UK; 13 Translational and Experimental Medicine, Division of Biomedical Sciences, Warwick Medical School, University of Warwick, Coventry, UK; 14 Centre of Applied Biological and Exercise Sciences, Faculty of Health and Life Sciences, Coventry University, Coventry, UK; 15 Department of Diabetology Endocrinology and Nephrology, Internal Medicine IV, University Hospital Tübingen, Eberhard Karls University Tübingen, 72074 Tübingen, Germany; 16 Division of Translational Diabetology, Institute of Diabetes Research and Metabolic Diseases (IDM) of the Helmholtz Center Munich, Eberhard Karls University Tübingen, 72074 Tübingen, Germany; 17 Department of Diabetes, School of Life Course Science and Medicine, Kings College London, London, UK; 18 Department of Internal Medicine IV, Gastroenterology, Hepatology and Infectious Diseases, Jena University Hospital, Jena, Germany

**Keywords:** Diseases, Molecular medicine, Immunology, Infectious diseases

## Abstract

We provide a descriptive characterization of the unfolded protein response (UPR) in skeletal muscle of human patients with peritoneal sepsis and a sepsis model of C57BL/6J mice. Patients undergoing open surgery were included in a cross-sectional study and blood and skeletal muscle samples were taken. Key markers of the UPR and cluster of differentiation 68 (CD68) as surrogate of inflammatory injury were evaluated by real-time PCR and histochemical staining. CD68 mRNA increased with sepsis in skeletal muscle of patients and animals (*p* < 0.05). Mainly the inositol-requiring enzyme 1α branch of the UPR was upregulated as shown by elevated X-box binding-protein 1 (XBP1u) and its spliced isoform (XBP1s) mRNA (*p* < 0.05, respectively). Increased expression of Gadd34 indicated activation of PRKR-Like Endoplasmic Reticulum Kinase (PERK) branch of the UPR, and was only observed in mice (*p* < 0.001) but not human study subjects. Selected cell death signals were upregulated in human and murine muscle, demonstrated by increased bcl-2 associated X protein mRNA and TUNEL staining (*p* < 0.05). In conclusion we provide a first characterization of the UPR in skeletal muscle in human sepsis.

## Introduction

Sepsis is characterized by organ dysfunction, mediated by a dysregulated host response to infection^1, 2^. Septic organ dysfunction may affect skeletal muscle and increases the risk for development of a persistent acquired muscle weakness syndrome in survivors, namely sepsis-induced myopathy^3^. In the long term, a substantial percentage of patients incompletely recovers from sepsis-induced myopathy, resulting in considerably impaired quality of life^4^. Sepsis-induced myopathy is characterized by reductions in force-generating capacity, impaired bioenergetics and muscle mass^3^. At the molecular level, myopathy has been linked to various aberrations including mitochondrial dysfunction, local effects of pro-inflammatory cytokines with consecutive degradation of myofibrillar proteins, upregulation of the proteasome proteolytic machinery, and altered protein synthesis^3^. Furthermore, acute insulin resistance and hyperglycemia have been linked to skeletal muscle dysfunction in septic patients^5–7^. However, understanding of the pathophysiology of sepsis-induced myopathy remains incomplete.

The endoplasmic reticulum (ER) represents a subcellular membranous network, essential for the maintenance of cellular processes^8^. Perturbations of ER homeostasis by inflammation or metabolic stress can induce cellular protein trafficking, resulting in a rise of folding defects and can provoke a condition called ER stress^8^. This initiates the activation of defined signaling cascades, namely the unfolded protein response (UPR)^8^. UPR sensor proteins like PRKR-Like Endoplasmic Reticulum Kinase (PERK) or inositol-requiring enzyme 1α (IRE1α) remain inactive when bound to glucose-regulated protein 78 kDa (also known as binding protein, BIP). Upon ER stress, BIP dissociates from its binding partners, resulting in subsequent activation of these sensor proteins. IRE1α activation leads to atypical splicing of X-box binding-protein 1 (XBP1u) mRNA into its transcriptionally active isoform (XBP1s), inducing increased expression of ER chaperones and activation of the ER-associated protein degradation pathway (ERAD). Under conditions of unresolvable ER stress induction of cell death represents an alternative strategy^8^. ER stress is an accepted contributor to pathologies defined by chronic low-grade inflammation and insulin resistance^8–10^. We hypothesized that ER stress could play a role in skeletal muscle under conditions of systemic major inflammation. As a highly conserved pathway the UPR could potentially link inflammation to sepsis-induced skeletal muscle weakness, as suggested by some animal models^11–13^. However, UPR activation has never been proven in skeletal muscle of sepsis patients so far. Therefore, we aimed to provide first *in human* data for the regulation of the UPR in skeletal muscle of septic patients and compare these findings to a complementary animal model.

## Results

### Patient characteristics

Baseline data of all subjects are given in Table 1 and baseline data of matched subgroups for mRNA analysis can be found in Suppl. Table 2. We found significant differences in terms of age, sex, body mass index (BMI), systemic inflammatory markers, kidney and liver function, and metabolic profile between the groups (Table 1; p < 0.05, respectively). Patients with sepsis were significantly older, had higher BMI and were more likely to be of male sex compared to the control group (p < 0.05). However, these figures were comparable between septic and insulin-resistant patients as these predefined subgroups were case-by-case matched for age and BMI (Table 1 and Suppl. Table 2; p > 0.05, respectively). Compared to insulin-resistant and control subjects, septic patients were characterized by significantly increased plasma markers of inflammation and impaired renal and hepatic function, reflecting sepsis-related organ dysfunction (Table 1; p < 0.05, respectively). Moreover, patients suffering from sepsis had increased homeostasis model assessment of insulin resistance (HOMA-IR), comparable to insulin-resistant subjects, and possibly reflecting both acute insulin resistance and inclusion of five patients (38%) with pre-existing type 2 diabetes (T2D). Since fasting blood samples were taken after surgery in the sepsis group, it cannot be excluded that surgical intervention per se could have contributed to elevated HOMA-IR, particularly, as HOMA-IR is a relatively vague estimate of insulin resistance in subjects suffering from sepsis. In support of an acute metabolic response, we found significantly reduced glucose transporter 4 (GLUT4) transcripts in septic skeletal muscle as compared to controls (p = 0.042; Suppl. Fig. 1). Otherwise, subjects characterized as chronically insulin-resistant had significantly elevated HbA1c levels when compared to both other groups, indicating the presence of chronic hyperglycemia (Table 1, Suppl. Table 2; p < 0.05, respectively). Control subjects did not show elevated markers of inflammation or insulin resistance (Table 1, Suppl. Table 2).

**Table 1:** Patient characteristics of the patient cohort.

Parameter	Control	IR	Sepsis	p-value
n (% male)	17 (35)	32 (69)	13 (57)	0.045^a^
Age [years]	55 ± 2	65 ± 1	69 ± 3	< 0.001^a,b^
BMI [kg/m^2^]	25.1 ± 1.0	28.4 ± 0.8	27.9 ± 1.1	0.028^a^
CRP [mg/l]	3.7 ± 0.6	12.4 ± 5.7	205.3 ± 27.5	< 0.001^b,c^
IL-6 [pg/ml]	4.9 ± 1.2	7.7 ± 1.3	623.8 ± 228.9	< 0.001^b,c^
White blood cell count [× 10^3^/µl]	6.0 ± 0.4	7.1 ± 0.3	17.2 ± 1.7	< 0.001^a,b,c^
Platelet count [× 10^3^/µl]	232.0 ± 16.3	255.5 ± 12.6	369.7 ± 45.9	0.032^b^
HbA_1c_ [%]	5.4 ± 0.1	7.0 ± 0.3	5.8 ± 0.3	0.001^a^
HbA_1c_ [mmol/mol]	36.1 ± 1.2	52.7 ± 3.4	39.6 ± 3.0	0.001^a^
HOMA-IR [AU]	1.5 ± 0.3	4.1 ± 0.9	4.8 ± 1.6	0.084
Fasting glucose [mmol/l]	7.0 ± 0.4	9.6 ± 0.4	7.1 ± 0.9	< 0.001^a,c^
Creatinine [µmol/l]	67.7 ± 2.6	88.9 ± 4.4	199.6 ± 33.2	< 0.001^a,b^
Albumin [g/l]	38.3 ± 0.7	37.3 ± 0.7	20.2 ± 1.4	< 0.001^b,c^
Bilirubin [µmol/l]	9.9 ± 1.3	9.8 ± 0.8	24.2 ± 5.8	0.011^b,c^
ALAT [µmol/l]	0.6 ± 0.1	0.6 ± 0.1	0.9 ± 0.2	0.55
γGT [µmol/l]	0.9 ± 0.2	1.6 ± 0.5	3.0 ± 0.7	0.08
Thromboplastin time [%]	108.1 ± 2.7	99.0 ± 3.9	77.2 ± 2.0	< 0.001^b,c^
Prior laparotomy n [%]	7 (41)	13 (40)	8 (61)	0.41
Malignancy n [%]	11 (65)	26 (81)	8 (61)	0.28
**Tumor localization [% total]**				
Breast	1 (9)	1 (4)	0 (0)	0.54
Colorectal	7 (64)	9 (35)	2 (25)	
Hepatobiliary	1 (9)	8 (31)	1 (12)	
Kidney	0 (0)	3 (12)	2 (25)	
Melanoma	0 (0)	1 (4)	1 (12)	
Pancreas	1 (9)	2 (8)	2 (25)	
Prostate	0 (0)	1 (4)	0 (0)	
Upper GI	1 (9)	1 (4)	0 (0)	
**Tumor disease stage [% total]**				
Local	3 (27)	14 (54)	7 (88)	0.03^b^
Advanced	8 (73)	12 (46)	1 (12)	
Prior chemotherapy n [%]	5 (29)	2 (6)	0 (0)	0.018*
Prior radiotherapy n [%]	4	1	0	0.021*

Data are given as Mean ± SEM or absolute numbers. Superscript letters indicate significant differences between groups (^a^control *vs*. IR, ^b^control *vs*. sepsis, ^c^IR *vs*. Sepsis, *post-hoc test not significant).

*ALAT* alanine-aminotransferase, *BMI* body mass index, *CRP* C-reactive protein, *GI *gastrointestinal tract, *GT* glutamyl-transferase, *Hb* hemoglobin, *HOMA-IR* homeostasis model assessment of insulin resistance, *IL* interleukin, *IR* insulin resistance; different superscript letters indicate p < 0.05 between subgroups.

More than half the patients suffered from underlying malignancies and groups were comparable in that regard (Table 1, Suppl. Table 2, p > 0.05). Notably, tumours were less advanced in septic patients compared to control and insulin-resistant subjects. Accordingly, patients in the control group were more likely to have received radio- or chemotherapy prior to study inclusion (Table 1).

The indications for laparotomy are given in Suppl. Table 3. Indications were similar between control and insulin-resistant subjects, except for primary hepatic or biliary hepatic malignancy, which were more abundant in the insulin resistant group. Naturally, indications for surgery were different in the sepsis group, ranging from insufficient anastomosis and gastrointestinal tract perforation to intraabdominal abscesses (Suppl. Table 3). About half the patients in all study groups had undergone previous open abdominal surgery (Table 1, Suppl. Table 2, p > 0.05).

### Skeletal muscle inflammation

**Figure 1:**
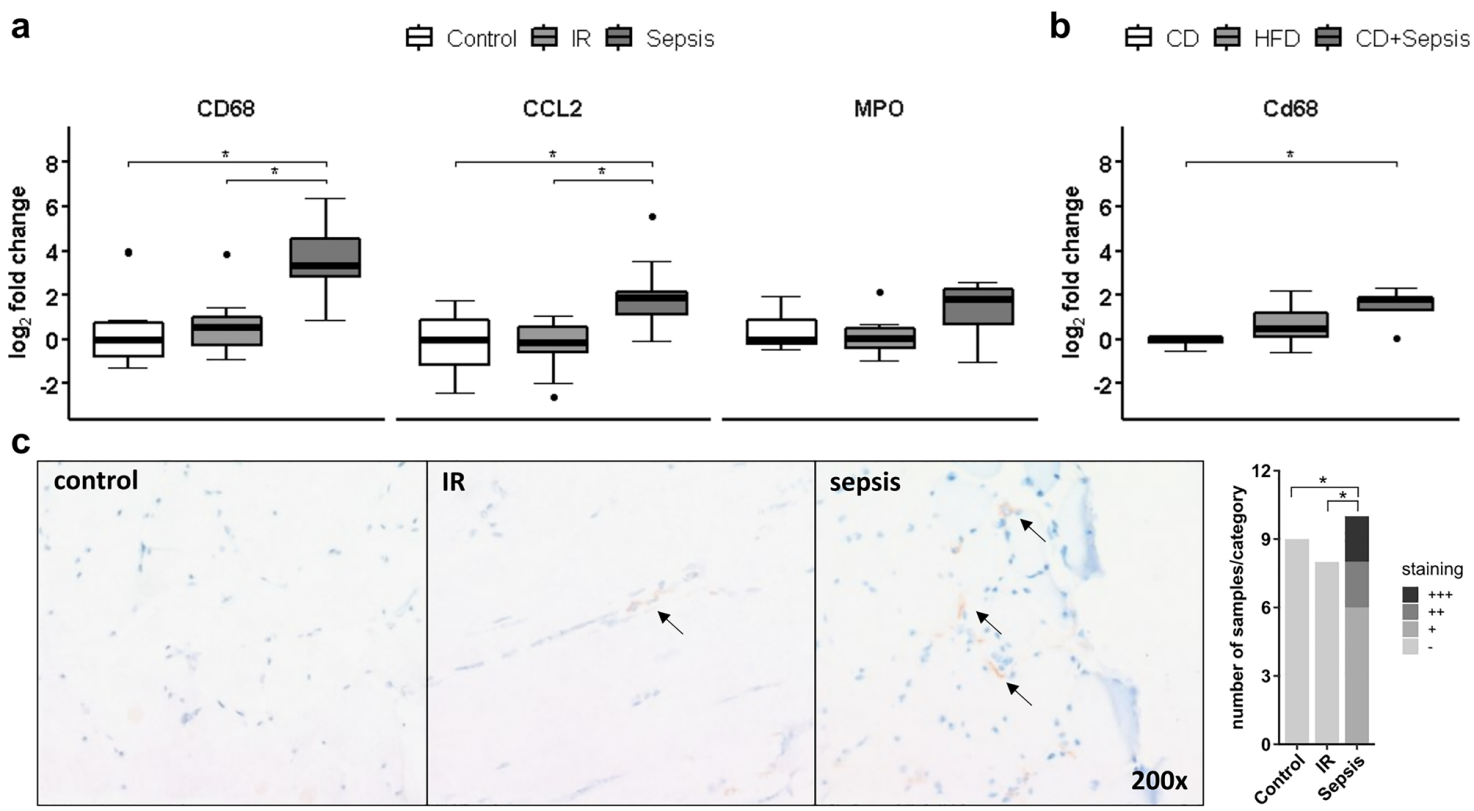
Markers of inflammation in skeletal muscle. Gene expression of inflammatory markers was assessed by RTqPCR in human (**a**) and murine (**b**) skeletal muscle. Data are given as normalized log_2_-fold changes compared to the median of the respective control groups as reference. Plots for human data represent n = 10 of matched subjects per group (for characterization see Suppl. Table 2), and plots for animal data represent n = 5–6 animals per group. Representative staining and semiquantitative assessment of CD68 in muscle tissue of patients (**c**) shows differences in number of macrophages (*p < 0.05, χ^2^-test). Arrows indicate exemplary CD68 positive cells (brownish coloring). *CD* control diet, *CD/Cd68* cluster of differentiation 68, *HFD* high-fat diet, *CCL* chemokine C–C motif ligand, *IR* insulin resistance, *MPO* myeloperoxidase, *RTqPCR* quantitative real-time PCR, *p < 0.05.

We identified skeletal muscle as a site of the systemic immune response to sepsis as demonstrated by significantly increased cluster of differentiation 68 (CD68) mRNA expression, a marker of local macrophage infiltration associated with skeletal muscle injury^14–18^, in human (Fig. 1a) and murine (Fig. 1b) sepsis (p < 0.05, respectively). Confirming local skeletal muscle inflammation, we found increased chemokine C–C motif ligand 2 (CCL2) transcripts in septic muscle (p < 0.05; Fig. 1a). Myeloperoxidase (MPO) and neutrophil elastase (ELANE) mRNA as indicators of neutrophil infiltration remained unchanged (p > 0.05, Fig. 1a and Suppl. Fig. 2, respectively).

To confirm increased CD68 in skeletal muscle on the protein level, we stained for CD68 in a subset of patients with sufficient quality of histological slides (n = 13, n = 19 and n = 11 patients in control, IR and sepsis groups, respectively) and found significantly increased CD68 staining in patients with sepsis compared to control and IR (p < 0.05, Fig. 1c).

### The UPR in skeletal muscle

**Figure 2:**
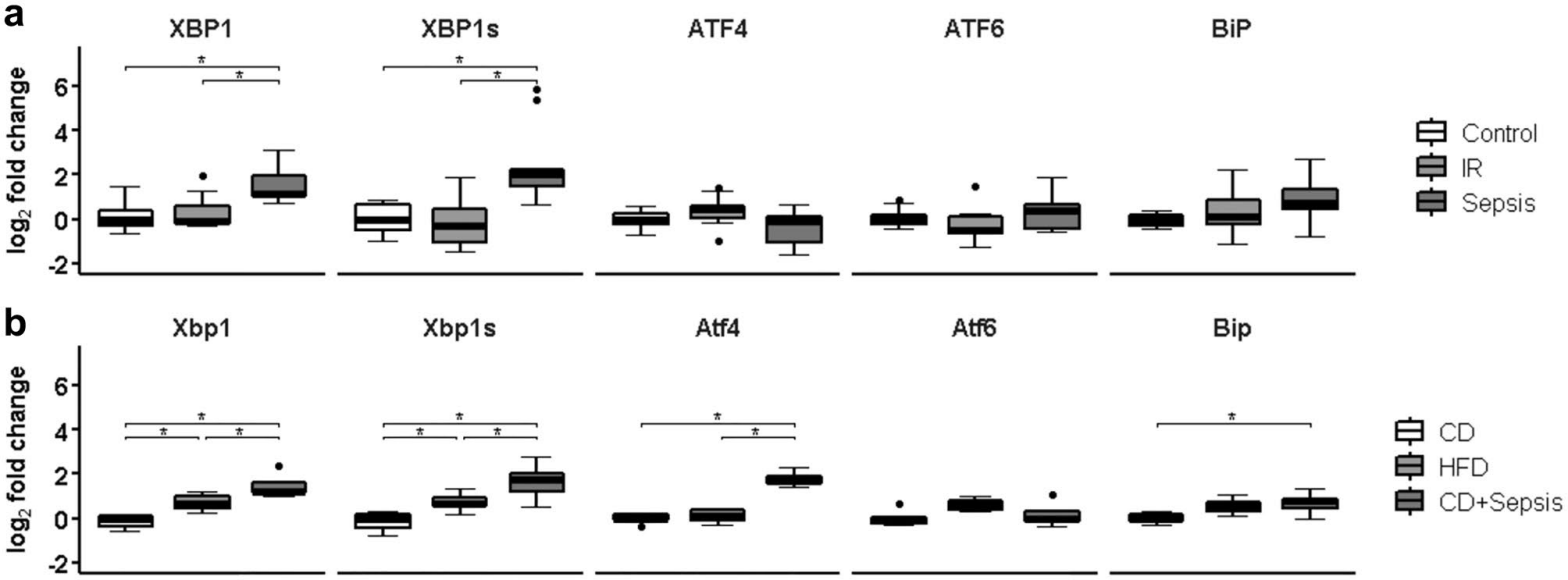
Gene expression analysis of indicators of UPR activation in human (**a**) and murine (**b**) skeletal muscle. Data are given as normalized log2-fold changes compared to the median of the respective control groups as reference. Plots for human data represent n = 10 of matched subjects per group (Suppl. Table 2), and plots for animal data represent n = 5–6 animals per group. *ATF* activated transcription factor, *BiP* binding protein, also known as glucose-regulated protein 78 kDa; *CD* control diet, *HFD* high-fat diet, *CD* control diet, *HFD* high-fat diet, *IR* insulin resistance, *XBP1s* X-box protein 1 spliced, *XBP1* X-box protein 1; *p < 0.05.

Markers of the UPR were up-regulated in sepsis compared to insulin-resistant or control conditions, as shown by activation of the highly conserved IRE1α branch (see Suppl. Fig. 3). Notably, we found a significant increase of XBP1u and XBP1s mRNA levels in skeletal muscle of both patients (Fig. 2a) and mice (Fig. 2b) suffering from peritoneal sepsis (p < 0.05, respectively). To confirm increased expression of XBP1 on the protein level, we performed immunohistochemical staining on a small subset of patients and observed more abundant nuclear localization in muscle tissue of septic patients (Suppl. Fig. 4). In contrast, mRNA of activating transcription factor (ATF) 4 and 6, representative of activation of both the other main branches of the UPR (Suppl. Fig. 3), were not increased in human skeletal muscle under either condition (p > 0.05, Fig. 2a), while Atf4 but not Atf6 mRNA was increased in murine peritoneal sepsis (p < 0.05 and p > 0.05; Fig. 2b). Neither mRNA of growth arrest and DNA damage-inducible protein 34 (GADD34; p = 0.76; Suppl. Fig. 5A) nor BIP (p = 0.42; Fig. 2b) were significantly regulated in human skeletal muscle. However, both were increased in murine peritoneal sepsis (p < 0.05, respectively; Fig. 2b and Suppl. Fig. 5b). Furthermore, the mRNA of c-Jun N-terminal kinase (JNK) was not significantly regulated in human skeletal muscle (p = 0.072; Suppl. Fig. 5c).

### Surrogate indicators of cell death

**Figure 3:**
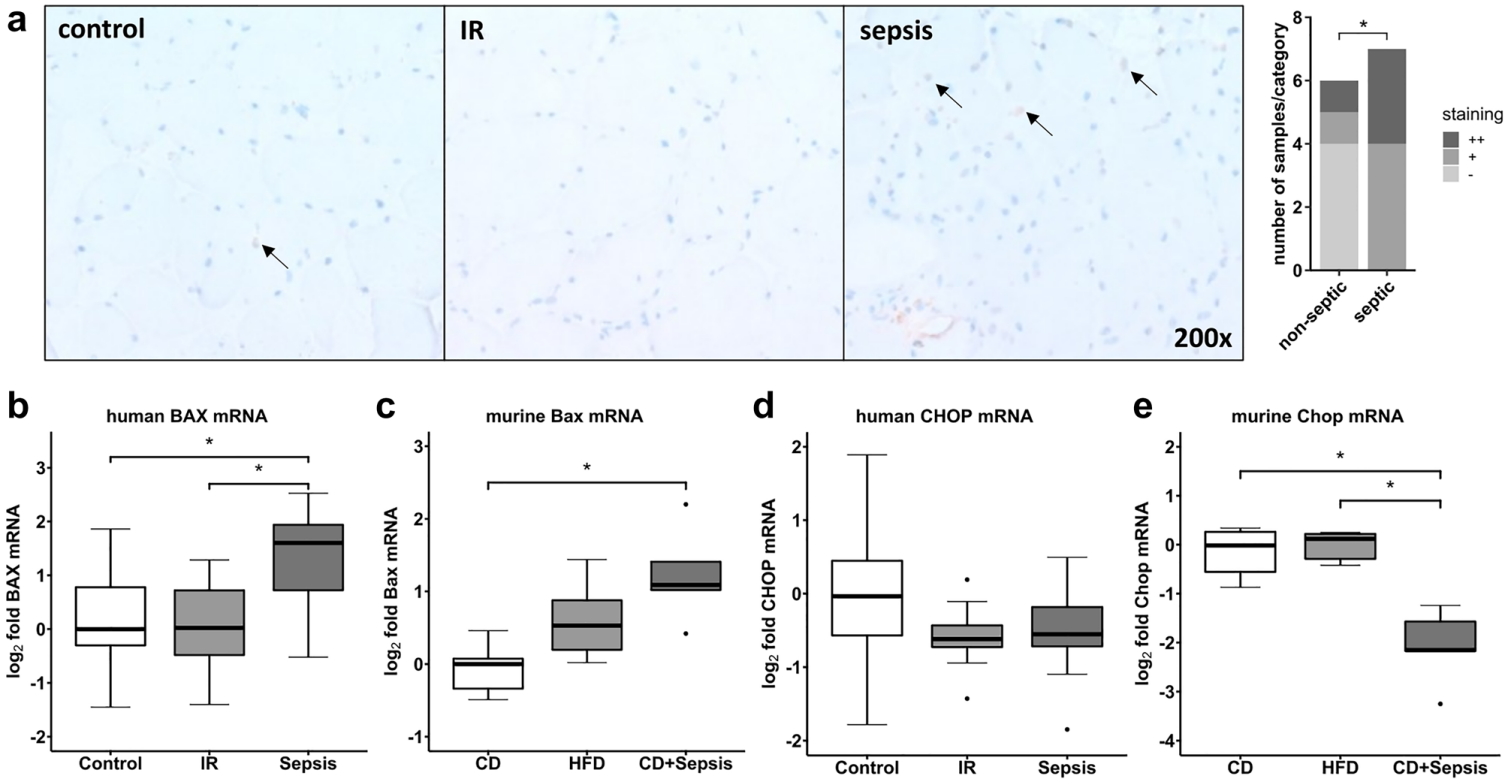
Indicators of apoptosis in skeletal muscle. TUNEL staining and semiquantitative assessment of positive cells (**a**) in human skeletal muscle in sepsis compared to the other groups (^*^p < 0.05, χ^2^-test). Arrows indicate TUNEL positive cells (brownish coloring), which are representative for cells undergoing apoptosis. Gene expression analysis for apoptotic marker genes BAX and CHOP, which are related to UPR activation, in human (**b**,**d**) and murine (**c**,**e**) skeletal muscle. Data are given as normalized log2-fold changes compared to the median of the respective control groups as reference. Plots for human data represent n = 10 of matched subjects per group (Suppl. Table 2), plots for animal data represent n = 5–6 animals per group. *BAX/Bax* bcl-2-associated X protein, *CD* control diet, *CHOP/Chop* C/EBP homologous protein, *HFD* high-fat diet, *IR* insulin resistance, *TUNEL* TdT-mediated dUTP-biotin nick end labeling; *p < 0.05.

We performed TdT-mediated dUTP-biotin nick end labeling (TUNEL) staining to identify cell death events in a small subset of non-septic controls and patients with sepsis (n = 6 and n = 7, respectively). TUNEL positive cells were more abundant in septic human muscle as compared to non-septic control subjects (Fig. 3a, p < 0.05), and not limited to nuclei corresponding to myotubes but also present in the interstitial space. Under septic conditions we further observed increased skeletal muscle mRNA of bcl-2-associated X protein (BAX) in both humans and animals (Fig. 3b,c; p < 0.05, respectively). By contrast, expression of C/EBP homologous protein (CHOP) remained without significant differences in patients (p > 0.05, Fig. 3d), but was significantly downregulated in murine skeletal muscle under septic conditions (p < 0.05, Fig. 3e). When staining for CHOP protein in human skeletal muscle by immunofluorescence, we did not find any difference in staining intensity (Suppl. Fig. 6).

### Cluster correlation analysis and Kaplan–Meier estimate

To further help the understanding of potential interactions in the UPR pathway in sepsis, we performed a cluster correlation analysis comprising mRNA transcripts of septic study patients (Suppl. Fig. 7a). CD68 transcripts were significantly correlated with the expression of CCL2 (r = 0.85; p < 0.001) and several UPR proteins, i.e. JNK (r = 0.63; p = 0.022), BIP (r = 0.80; p = 0.019) and ATF6 (r = 0.70; p = 0.007). Furthermore, we found a positive association of CD68 mRNA with BAX (r = 0.57, p = 0.041). No significant correlation became apparent with XBP1u (r = 0.32, p = 0.29), XBP1s (r = 0.29; p = 0.33), ATF4 (r = 0.04; p = 0.90), or GADD34 (r = 0.40; p = 0.20). Supplementary Fig. 7b depicts the cluster correlation for murine septic skeletal muscle for comparison with distinct differences in clustering of transcripts. Cluster correlation matrices for control and insulin resistant human and murine skeletal muscle mRNA expression can be found in Suppl. Fig. 8.

**Figure 4:**
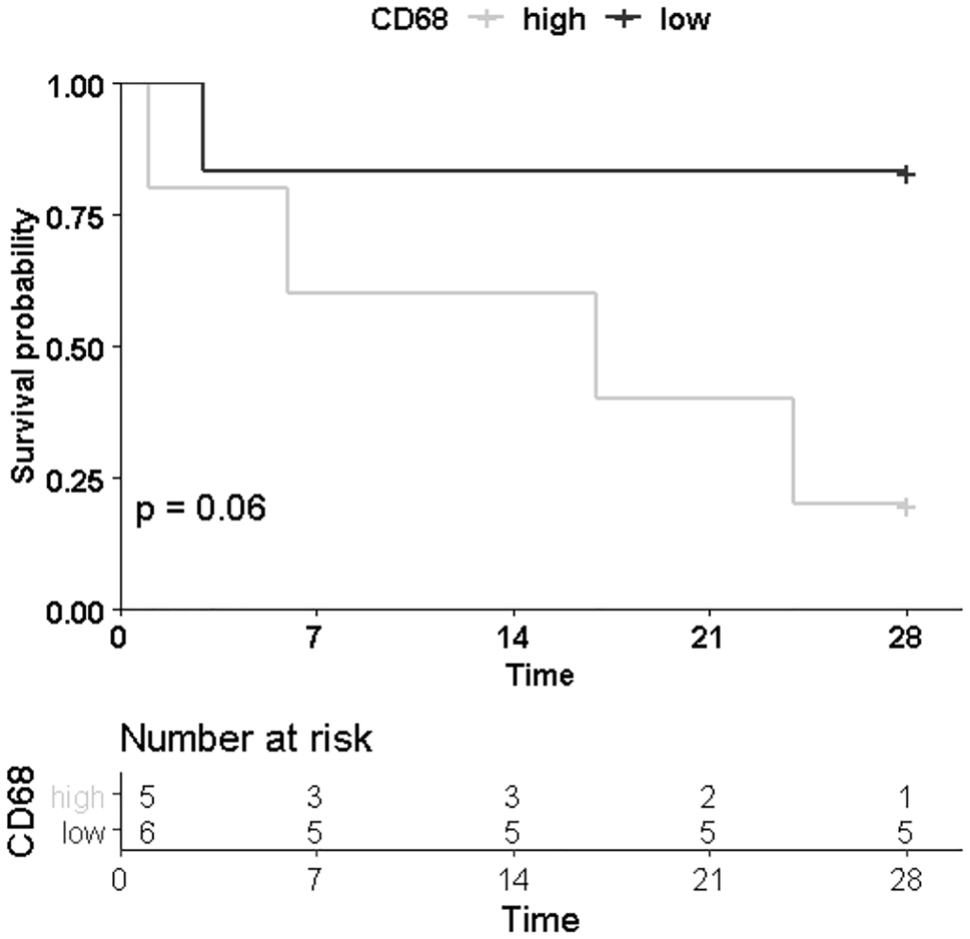
Kaplan–Meier estimated curve of 28-day survival of patients suffering from peritoneal sepsis based on CD68 immunostaining in human skeletal muscle. 28-day survival was lower in patients with high number of infiltrating CD68 positive cells but not statistically significant in log-rank test (p = 0.06). CD68, cluster of differentiation 68.

To further evaluate whether skeletal muscle inflammation may be associated with patient outcome, we performed an explorative post-hoc Kaplan–Meier analysis, studying patient survival in relation to the abundance of CD68^+^ cell infiltration in skeletal muscle. However, mortality was not significantly different in patients with more infiltrating cells compared to those with lower numbers (5/6 vs. 2/5 patients, respectively; p = 0.06; Fig. 4), probably owing to low number of subjects and inadequate power.

## Discussion

We provide novel findings showing that peritoneal sepsis, in humans and mice, is related to skeletal muscle macrophage infiltration and local activation of the unfolded protein response.

Animal models suggest a critical role of ER stress in sepsis^11, 13^. In support of this, a recent study in septic shock patients revealed associations of plasma mRNA of defined ER stress markers with organ failure^19^. Moreover, the group of Jiao et al. was able to show in an endotoxin model that key proteins of the UPR, such as Bip and Chop, increase in rat diaphragm with systemic inflammation and this was accompanied by weakened muscle contractile force^13^. Therefore, it was discussed that the ER stress pathway could represent a novel target in the therapy of inflammatory myopathies^12, 20^. Our data provide evidence for the presence of a local inflammatory co-response in skeletal muscle with systemic high-grade inflammation. The latter is supported by macrophage infiltration and increased expression of CD68 mRNA in skeletal muscle of subjects suffering from peritoneal sepsis. We therefore expand the findings of Jiao et al.^13^, showing a significant relationship of the inflammation surrogate marker CD68 with the expression of UPR indicators in muscle tissue. CD68 is an indicator of macrophage tissue infiltration and macrophages have been identified as pivotal regulators in the skeletal muscle response to tissue injury^21, 22^. However, local inflammation and macrophage infiltration are two-edged swords with the potential to both enhance injury but also initiate and promote tissue regeneration^23^. We did not assess the macrophage polarization state and therefore, whether macrophage infiltration at this stage of human sepsis primarily represents a more maladaptive or regenerative response needs to be elucidated in future studies. Moreover, with our study design we cannot conclude a causal relationship. From our data it can only be hypothesized that the local inflammatory response to sepsis in skeletal muscle contributed to ER stress and thereby elicited the UPR^12, 20^.

Our findings of increased HOMA-IR and reduced GLUT4 expression point towards sepsis-induced insulin resistance^6^. ER stress has been shown to be related to reduced tissue glucose uptake, and it was demonstrated that diabetes can be treated by using novel pharmacologic UPR modulators^24^. Therefore, metabolic perturbations could have played an alternative or synergistic role in UPR activation in our subjects since skeletal muscle is known as major site of insulin action. The activating effect on the highly conserved IRE1α branch of the UPR was observed exclusively in septic study subjects, but not in chronically insulin-resistant controls. Our findings in patients and animals therefore suggest that mainly local macrophage infiltration and not hyperglycemia/insulin resistance were related to muscle UPR induction.

Activation of IRE1α can promote JNK activity and boost inflammatory responses^12^. The latter is supported by our correlational analysis, showing associations of CD68 with pro-inflammatory transcripts. Under local inflammatory conditions the primary role of IRE1α activation is the restoration of protein homeostasis and cellular function by boosting ER folding capacity^12^. Another effect is reduction of general protein synthesis to decrease ER stress by reducing the load of un-/misfolded protein^20^. However, with unresolvable ER stress, induction of cell death represents an alternative strategy^8^. We found significant correlations of CD68 with BAX as an indicator of apoptosis. This observation correlates with the findings of Jiao et al.^13^ and evolves the hypothesis of an effect of local inflammation on cell death induction in skeletal muscle. Activated JNK can induce CHOP expression and thereby initiate apoptosis^12, 20^. We found significantly increased mRNA expression of BAX in septic muscle, which was in accordance with the findings on cell death of our TUNEL analysis. By contrast, CHOP protein remained unaltered in septic patients. This, however, is in accordance with recent findings showing unchanged CHOP expression in human plasma after five days of septic shock^19^. By contrast, CHOP was downregulated in skeletal muscle of our septic rodent model, while GADD34 was increased. We can only speculate on the reason for this difference between human and murine skeletal muscle response to sepsis, but a possible explanation could be a different time point of tissue sampling in the course of the disease. Nevertheless, we need to acknowledge that apoptosis is a complex process, involving several different activating pathways and posttranslational protein modifications—a complexity that is not well reflected by our limited selection of markers. Furthermore, positive TUNEL staining was not limited to nuclei of myotubes but also extended to interstitial cells, likely satellite cells, endothelium, or immune cells, which probably have contributed to the observed effects. Therefore, we can finally only state from our data that the local inflammatory response to sepsis was correlated with UPR activation and increased cell death in skeletal muscle, but we cannot deduce a causal relationship.

Accelerated organ inflammation can be related to a more accentuated course of sepsis. Therefore, we performed an explorative Kaplan–Meier analysis looking for a difference in survival between patients with increased CD68 positive cells in skeletal muscle histology compared to patients with low numbers. We found no significant differences between groups, probably owing to the low number of patients. Notably, as we did not plan this analysis a priori, the survival analysis was inadequately powered, limiting the validity of survival estimates. Larger cohorts would be necessary to adequately assess the association of skeletal muscle inflammation and UPR activation with prognosis in patients with sepsis. Existing data on the presence of local macrophage infiltration are conflicting as some found CD68 positive cells in skeletal muscle of critically ill patients with related myopathy, associated with necrotic cell death^18^, while others did not^25^. In contrast to others, we included only subjects suffering from peritoneal sepsis and thus, studied a relatively homogenous sample. This could have contributed to explain varying findings. Future studies will need to confirm our findings of increased macrophage infiltration and apoptosis in skeletal muscle of septic subjects.

Limitations of the current study are its observational nature and therefore, we cannot directly address mechanisms or causal links. Moreover, our results on the UPR in human skeletal muscle could have been influenced by a potential selection bias, as a variety of pathologies can induce ER stress. Specifically, both insulin resistant and septic patients were older, had higher BMI and a higher percentage of males compared to control subjects, potentially contributing to the observed UPR activation and skeletal muscle inflammation. However, insulin resistant and septic patients were comparable in that regard and therefore, it is unlikely that differences in UPR transcript expression between these groups are attributable to differences in patient demographics. Moreover, controls and patients with peritoneal sepsis showed comparable values for HbA1c as an indicator for chronic glycemic status whereas both insulin resistant and septic patients had increased HOMA-IR, reflecting more acute insulin resistance in these groups.

Malignancies are another potential biasing factor, and increased activation of skeletal muscle UPR has been described in mouse models of cancer cachexia^26^. Notably, tumor stages were different between study groups and a small proportion of patients in the control and IR group but not in sepsis received prior cancer treatment, which could have affected results. As tumors were less advanced in patients with peritoneal sepsis, we believe that tumor-associated muscle wasting is unlikely to have contributed to increased expression of UPR markers in sepsis, as we would expect higher levels of UPR in patients with more advanced disease and cancer treatment.

We were able to replicate our main findings of UPR activation and apoptosis in a mouse model of peritoneal sepsis. We chose the peritoneal contamination and infection (PCI) model of peritoneal sepsis because it is reproducible, easy to perform, largely reflects human disease^27, 28^ and is well established in our institute. Other models of peritoneal sepsis, like coecal ligation and puncture (CLP), autologeous PCI or peritoneal injection of bacteria or LPS might produce different results, owing to surgical wounds, different microbial composition or the absence of human-derived material and cells and would be worth exploring in the future. Limited transferability of animal data into the clinical setting remains a general concern in experimental research^29^, warranting further investigation in clinical sepsis cohorts.

Our results are limited to human *rectus abdominis* and murine *gastrocnemius* muscle. It is well established that different types of skeletal muscle show distinct gene expression patterns^30^, which might partly explain the observed differences between human and mouse data and limits the generalizability of our results to other muscle groups. Moreover, animal specimens were taken strictly 24 h after sepsis induction and patient samples were not taken later than 24 h after diagnosis of sepsis, but most patients with sepsis were already hospitalized for a couple of days. This means that patients might have been in a state of inflammation for a longer time before tissue sampling, introducing a potential bias in the analyses. Adding to this, despite patients with abdominal surgery in the preceding 5 days were excluded, about half the patients had undergone previous laparotomy, and scarring and wound healing processes might have distorted results. However, the proportion of patients with prior laparotomy was comparable between study groups, rendering a systematic bias unlikely. Furthermore, limited size of tissue specimen prevented quantification of protein levels and the study of phosphorylation and cleavage events involved in UPR activation. Therefore, activation of the PERK and ATF6 axes, including ATF4, might have been missed, as posttranslational modifications are main events in activation of these pathways^8^. Likewise, small size of tissue specimen and low RNA yield prevented analyses of additional transcripts to underpin our observations and make more detailed assumptions of the exact pathways involved. Moreover, due to the lack of data and low number of subjects we cannot provide results on clinical outcomes regarding skeletal muscle function and long-term morbidity. This and the potential effect of UPR-directed therapies need to be subject of future investigations. Finally, our data are exclusively representative for Caucasian subjects and patients suffering from peritoneal sepsis and cannot be generalized to other ethnicities or septic entities.

In summary, we provide first evidence for a co-activation of local inflammation and the IRE1α branch of the UPR in skeletal muscle in response to peritoneal sepsis. Our findings could be of clinical significance for ICU acquired muscle weakness, as ER stress potentially contributes to its pathophysiology. Future investigations will need to elucidate the exact role of ER stress and the UPR as a potential therapeutic target.

## Materials and methods

### Study design

This study aimed to characterize the UPR and its potential associations with molecular surrogates of local injury in skeletal muscle of septic patients in a clinical study. For this purpose, we investigated blood and skeletal muscle samples from a previous human study including patients with peritoneal sepsis undergoing therapeutic laparotomy^31^. The CD68, an accepted indicator of macrophage infiltration in response to skeletal muscle injury^14–18^, was selected as key surrogate of muscular inflammatory response to systemic inflammation.

The UPR is a recognized driver in pathologies defined by chronic low-grade inflammation and insulin resistance^9^, and thus, we included patients with insulin-resistance, but without concurrent sepsis as “positive controls”. Patients without clinical evidence of both sepsis and insulin resistance were included as “negative controls”. All patients in both control groups underwent median laparotomy for medical indication as well. For intergroup comparison analyses, we included subgroups with ten patients each, case-by-case matched for age and BMI in septic and insulin resistant subjects.

We collected baseline data from standard laboratory and clinical measurements. Gene expression of representative transcripts of the UPR and inflammatory response pathways were assessed by qRTPCR. Histochemical and immunohistochemically staining was performed in all subjects. An established murine model of polymicrobial peritoneal sepsis was studied for complementary confirmation^31^.

### Subjects and ethics

This investigation was a secondary analysis of samples gained in a previous study^31^. The study protocol was approved by the faculty’s ethics review board of the Jena University Hospital (3247-09/11) and conformed to the ethical guidelines of the 1975 Declaration of Helsinki. All methods were performed in accordance with relevant guidelines and regulations. All subjects or their legal representatives gave written informed consent. We included patients undergoing therapeutic laparotomy aged 18 years or older, that have had not undergone other surgical interventions during five days prior to enrolment. As reported earlier, subjects in the sepsis group met criteria for diagnosis of sepsis following criteria of the guidelines of the German Sepsis Society^32^. We included patients into the insulin resistance group if they had known T2D with antidiabetic therapy, fulfilled the criteria of the American Diabetes Association for T2D or the National Cholesterol Education Adult Treatment Panel III criteria for metabolic syndrome^33, 34^. Antidiabetic medication, if applicable, was discontinued before surgery. Control subjects did not match criteria for diagnosis of sepsis or insulin resistance. General exclusion criteria were chemotherapy within the last two months, long-term immune-suppressive treatment, history of organ transplantation, active rheumatoid inflammatory disease, drug or alcohol abuse (defined as a daily alcohol intake of more than 20 g for females and 40 g for males), pre-existing chronic kidney disease or kidney failure with essential hemodialysis and known liver cirrhosis.

### Human blood and tissue samples

Insulin-resistant subjects and surgical controls underwent an overnight fast and peripheral blood was taken in the morning before laparotomy. Due to logistic reasons arising from emergency surgery in patients with abdominal sepsis, blood from these subjects was obtained on the morning of the day after surgery to get blood after a fasting period of at least eight hours. All clinical laboratory parameters were measured in certified university hospital laboratories^9^. Whole-body insulin resistance was estimated by HOMA-IR^35^. Skeletal muscle tissue samples were taken right after midline incision and preparation from the M. rectus abdominis, and immediately snap frozen in liquid nitrogen and stored at − 80 °C^36^. A small part was directly separated and stored in a humidified chamber for histological analysis until it was embedded in Tissue-Tek® O.C.T.TM Compound (Sakura Finetek, Japan) and immediately stored at − 80 °C. Blood samples remained on ice until centrifugation. Serum was prepared by centrifugation with 3000×*g* for 10 min at 4 °C and then stored at − 80 °C.

### Animal model

We performed all investigations and experiments in accordance with the German legislation on protection of animals and obtained permission to conduct the study from the regional animal welfare committee of the Friedrich-Schiller-University in Jena (registration number 02-038/12)^31^. All methods were carried out in accordance with relevant guidelines and regulations, and are reported in accordance with the ARRIVE guidelines.

Six-week-old male C57BL/6J mice were kept under standardized laboratory conditions and fed a standard chow (control diet, CD) or a diet containing 34% of fat (high-fat diet, HFD, ssniff EF R/M D12492 mod.) for 12 weeks. The HFD was used as a model of obesity-induced insulin resistance^31^. Weight was measured every two weeks to ensure adequate feeding and body mass development. After 12 weeks, CD animals were randomized to undergo PCI procedure to induce peritoneal sepsis, resulting in three groups of mice: CD baseline (CD), HFD baseline (HFD), CD sepsis (CD24h).

To induce sepsis, we used the peritoneal contamination and infection model^31^, administering a standardized diluted human stool slurry (1.25 µL/g body weight) intraperitoneally. After 12 h of sepsis, mice received 20 µL/g body weight saline solution subcutaneously to mitigate the impact of dehydration. At baseline or 24 h after sepsis induction, mice were killed by taking citrate-anticoagulated blood by cardiac puncture under deep isoflurane anesthesia. Blood samples were kept on ice and plasma was produced by centrifugation at 2500×*g* for 10 min at 4 °C. Skeletal muscle samples were taken as one complete *M. gastrocnemius* from each animal and immediately snap frozen in liquid nitrogen and stored at − 80 °C until measurements. The contralateral gastrocnemic muscle was embedded in Tissue-Tek® O.C.T.TM Compound (Sakura Finetek, Japan) and directly stored at − 80 °C for histology.

### Skeletal muscle mRNA expression

We extracted mRNA with Trizol isolation using Qiazol Lysis Reagent and a bead mill. RNA had to be concentrated by vacuum centrifugation and concentrations were measured by spectrophotometry with NanoDrop 1000 (PeqLab, Germany). RNA integrity was tested with automated electrophoresis with Experion Automated Electrophoresis System (Bio-Rad Laboratories, USA). Next, RNA was transcribed into cDNA with RevertAid First Strand cDNA Synthesis Kit (Fermentas, USA). PCR reactions were performed on Rotor-Gene Q (QIAgen, Germany) in a total reaction volume of 20 μL with Brilliant II SYBR Green qPCR Master Mix (Stratagene, USA) and forward and reverse primers (Biomers, Germany). Expression analyses were performed as reported^31^. Primer sequences and complete methods are given in the supplemental section (supplementary methods and Suppl. Table 1).

### Histological assessment and staining

Skeletal muscle tissue slices of 5 µm thickness were produced by cryosectioning with Leica CM3050S cryostat (Leica Biosystems, USA) and refrozen at − 80 °C. After quality control with Haematoxylin–eosin (H&E) staining, only muscle slides of high muscle fiber quality were used for further staining. H&E and XBP1-staining (anti-XBP1-antibody, ab37152, abcam, United Kingdom) were performed as described previously^9^. CD68 skeletal muscle staining was performed using a PG-M1 antibody (Dako Cytomation, Germany) and the Dako REAL Detection System (LSAB +) HRP/DAB + (Dako Cytomation) and was counterstained with haematoxylin. TUNEL-staining was performed using the In Situ Cell Death Detection Kit POD (Roche Deutschland Holding GmbH, Germany). Methods are described in more detail in the supplement.

Microscopic analyses were performed with the Olympus Provis AX 70 microscope (Olympus, Germany) and the Nikon Eclipse Ti-E with X-Cite®-fluorescence model (Nikon, Japan). Staining for CD68, XBP1 and TUNEL staining were scored based on the apparent intensity and abundance of staining by a blinded investigator (UBM) on an ordinal scale from zero to three, indicating no, minimal, moderate, and high staining intensity.

### Statistical analysis

SPSS 22.0 (SPSS Inc., USA) and R version 3.6.2 in RStudio (RStudio Inc, USA) were used to perform statistical analyses. Graphs were drawn using the R packages *ggpubr* and *ggplot2*.

Data are given as means ± SEM, if not stated otherwise. Boxes in all boxplots span from 25th to 75th percentile, whiskers indicate minimum and maximum values inside the 1.5 times interquartile range above and below first and third quartiles, and outliers are depicted as dots. Normal distribution was tested with Shapiro-Wilks-Test. To test for homogeneity of variance we used the Levene procedure. Depending on data distribution, we used the following statistical procedures: One-way analysis of variance (ANOVA), Welch’s test or Kruskall-Wallis test with post hoc Bonferroni-Holm or Dunn-Holm correction, and χ^2^-test for testing of ordinal or nominal data. Heatmaps for clustered correlation matrices were made with the R package *heathmaply* using Spearman correlation coefficients and complete-linkage hierarchical clustering. The Kaplan–Meier method was used to study the survival function of sepsis patients, and significance was evaluated by using a log-rank test. An alternative hypothesis was accepted if two-sided p < 0.05.

### Ethics statement

The studies involving human participants were reviewed and approved by Ethics Committee of the Friedrich Schiller University Jena. The patients/participants provided their written informed consent to participate in this study.

## Supplementary Information

Supplementary Information.

## Data Availability

The datasets generated for this study are available on reasonable request to the corresponding author.

## Abbreviations

ACTB: β-Actin
ASK1: Apoptosis signal regulating factor 1
ATF4: Activating transcription factor 4
ATF6: Activating transcription factor 6
BAX: Bcl-2-associated X protein
BIP/GRP78: Binding immunoglobulin protein / glucose-regulated protein 78
BMI: Body Mass Index
CCL2: Chemokine C–C motif ligand 2
CD68: Cluster of differentiation 68
CHOP: C/EBP homologous protein
CLP: Cecal ligation and puncture
CRP: C-reactive protein
eIF2α: Eukaryotic initiation factor 2α
ELANE: Neutrophil elastase
ER: Endoplasmic reticulum
ERAD: ER-associated protein degradation
GADD34: Growth arrest and DNA damage-inducible protein 34
GAPDH: Glyceraldehyde-3-phosphate dehydrogenase
GLUT4: Glucose transporter 4
HbA1C: Glycated hemoglobin A1c
HFD: High-fat diet
HMBS: Hydroxymethylbilan synthase
HOMA-IR: Homeostasis model assessment of insulin resistance
HPRT: Hypoxanthin-phosphoribosyltransferase
IL-6: Interleukin-6
IRE1: Inositol-requiring-enzyme 1
JNK/MAPK8: C-Jun N-terminal kinase/mitogen-activated protein kinase 8
kDa: Kilo-Dalton
MPO: Myeloperoxidase
PBS: Phosphate buffered saline
PCI: Peritoneal contamination and infection
PERK: PRKR-Like Endoplasmic Reticulum Kinase
TRAF2: Tumor necrosis factor receptor-associating factor 2
TUNEL: TdT-mediated dUTP-biotin nick end labeling
T2D: Type 2 diabetes mellitus
UPR: Unfolded protein response
XBP1s: X-box binding-protein 1, spliced variant
XBP1u: X-box binding-protein 1, unspliced variant
