# Supplementary material for: Endoplasmic reticulum stress and the unfolded protein response in skeletal muscle of subjects suffering from peritoneal sepsis
Source: Sci Rep. 2022 Jan 11;12:504. doi: 10.1038/s41598-021-04517-9 (PMC8752775; doi:10.1038/s41598-021-04517-9)
Supplement: Supplementary file 1 — Supplementary Information. [file 41598_2021_4517_MOESM1_ESM.pdf]

I. Supplementary Methods

Skeletal Muscle Tissue mRNA Expression

Tissue samples were milled with Qiazol Lysis Reagent (Qiagen GmbH) and a metallic ball in a ball mill at 30 Hz for four minutes to disrupt tissue and membranes. 200 µl of chloroform (Sigma-Aldrich Chemie GmbH, Germany) were added, mixed, and centrifuged at 12,000 x g for 20 minutes at 4 °C. The remaining liquid phase was transferred into a sterile tube and 550 µl isopropanol (Sigma-Aldrich Chemie GmbH, Germany) and 80µl sodium acetate (2M, pH=4,0; Sigma-Aldrich Chemie GmbH, Germany) were added. Tubes were frozen at -80 °C for 24 hours and after defrosting were centrifuged again to eliminate the liquid upper phase. The remaining pellet was resuspended in ethanol (Nordbrand Nordhausen GmbH, Germany). Due to low RNA yield, we had to concentrate samples by centrifugation and drying in vacuum, followed by resuspending the pellet in 25 µl of nuclease-free water (Carl Roth GmbH+Co KG, Germany). RNA was stored at -80°C until further processing.

RNA concentrations were measured by spectrophotometry with NanoDrop 1000 (PeqLab, Germany). RNA Integrity was tested using automated electrophoresis with Experion Automated Electrophoresis System (Bio-Rad Laboratories, USA). Reversal transcription into cDNA was performed by using RevertAid First Strand cDNA Synthesis Kit (Fermentas, USA). 10 µl Brilliant II SYBR Green qPCR Master Mix (Stratagene, USA) were mixed with 5 µl of 5 µM forward and reverse primers (Biomers, Germany) and 5 µl of cDNA with a concentration of 5 ng/µl. This led to a total reaction volume of 20 µl. RT-qPCR was performed on Rotor-Gene Q (QIAgen, Germany). Relative expression for each primer was interpolated from standard

curves. Following reference gene analysis with GeNorm (Vandesompele et al. 2002) and NormFinder (Andersen et al. 2004), means of relative expression of β-Actin, glyceraldehyde-3-phosphate dehydrogenase, hypoxanthine phosphoribosyltransferase, and porphobilinogen deaminase were calculated and used as normalization factors. Normalized gene expression is given as $\log_2$-fold expression of the gene of interest relative to the median of expression of the control group. Primer sequences are given in the supplemental section.

Histological Assessments and Stainings

Skeletal muscle tissue slices of 5 µm thickness were produced by cryosectioning with Leica CM3050S cryostat (Leica Biosystems, USA) and refrozen at -80 °C. After quality control only muscle slides of high muscle fibre quality were used for further staining. Haematoxylin-eosin and XBP1-staining (anti-XBP1-antibody, ab37152, abcam, Great Britain) were performed. CD68 skeletal muscle staining was performed using the PG-M1 antibody (Dako cytomation, Germany), which detects the macrophage-restricted form of CD68 (PMID: 7684194), and the *Dako REAL™ Detection System (LSAB+) AP/RED* (Dako cytomation). Counterstaining was performed with haematoxylin.

The TdT-mediated dUTP-biotin nick end labeling (TUNEL)-staining was performed using the In Situ Cell Death Detection Kit POD (Roche Deutschland Holding GmbH, Germany). Slices were defrosted for one hour at room temperature, dried, fixed in 5 % formalin, washed in PBS and distilled water for five minutes. To block endogenous peroxidase activity slices were incubated in a dilution of 3 % hydrogen peroxide in methanol (Carl Roth GmbH and Co KG, Germany) and then washed three times with

PBS and water. A mixture of reaction buffer and Terminal deoxynucleotidyl transferase (TdT) was prepared and 60 µl of the mixture were added, followed by one hour of incubation at 37 °C in a humidified chamber. After washing with PBS, 60 µl of the Anti-Converter POD were added and slices were incubated for 30 minutes at 37 °C.

Microscopic analysis of the haematoxylin-eosin, the CD68, the XBP1 and the TUNEL-stainings were performed with the Olympus Provis AX 70 microscope (Olympus, Germany). Microscopic analysis of the CHOP staining was performed at the fluorescence microscope Nikon Eclipse Ti-E with X-Cite®-fluorescence model and the equipped DS-Qi2-camera (Nikon, Japan).

Suppl. Table 1: Primer sequences for skeletal muscle mRNA expression analysis.

**Human Gene**	**5'-3'-Sequence**
ACTB	f: ggc atg ggt cag aag gat t<br>r: agg tgt ggt gcc aga ttt tc
GAPDH	f: ctc tgc tcc tcc tgt tcg ac<br>r: caa tac gac caa atc cg ttg ac
HMBS	f: atg tct ggt aac ggc aat gc<br>r: cgt ctg tat gcg agc aag c
HPRT	f: cct ggc gtc gtg att agt gat<br>r: aga cgt tca gtc ctg tcc ata a
ATF4	f: gtt ctc cag cga caa ggc taa<br>r: gca tcc tcc ttg ctg ttg ttg
ATF6	f: cct tca gcg aat agc cca gt<br>r: gca cag caa tat ctg aac cga c
BIP	f: acc gct gag gct tatt tgg g<br>r: gtc ttt ggt tgc ttg gcg tt
BAX	f: gtc gcc ctt ttc tac ttt gcc<br>r: cgg agg aag tcc aat gtc c

CCL2	f: cat agc agc ctt cat tcc<br>r: tct gca ctg aga tct tcc tat tgg
CD68	f: gct aca tgg cgg tgg agt aca a<br>r: atg atg aga ggc agc aag atg g
CHOP	f: gag ctg gaa gcc tgg tat ga<br>r: gac ggg tca aga gtg gtg aa
GADD34	f: ctg gct ggt gga agc agt aa<br>r: tat ggg gga ttg cca gag ga
GLUT4	f: ctg tgg ctg gtt tct cca act<br>r: gag gac cgc aat aga agg aag
ELANE	f: cgt ggc gaa tgt aaa cgt cc<br>r: tta cgg ggt cgt agc cgt t
JNK	f: gat gct gtg tgg aat caa gca c<br>r: ggc cag acc gaa gtc aag aat
MPO	f: ccg gga tgg tga tcg gtt tt<br>r: gtg gtg atg cct gtg ttg tc
XBP1u	f: gtg agc tgg aac agc aag tgg t<br>r: cca agc gct gtc tta act cct g
XBP1s	f: gtg agc tgg aac agc aag tgg t<br>r: ctg cac cct gct gcg gac t
**Mouse Gene**	**5'-3'-Sequence**
Actb	fw: gct ctt ttc cag cct tcc tt<br>rv: cgg atg tca acg tca cac tt
Gapdh	fw: caa cag caa ctc cca ctc ttc<br>rv: ggt cca ggg ttt ctt act cctt
Hmbs	fw: gaa atc att gct atg tcc acc a<br>rv: gcg ttt tct agc tcc ttg gta a
Atf4	fw: tcg gcc caa acc tta tga cc<br>rv: tgg ctg ctg tct tgt ttt gc
Atf6	fw: tgg gaa tgg aag cct aaa gag g<br>rv: cgt ggg agg aca gag aaa ca
Bax	fw: atg cgt cca cca aga agc tg<br>rv: cgt cag caa tca tct gc
Bip	fw: cac cag gat gcg gac att ga<br>rv: cca ctt cca tag agt ttg ctg a
Chop	fw: tct tga gcc taa cac gtc gat<br>rv: tcc ttc tgg aac act ctc tcc t
Cd68	fw: agg acc gct tat agc cca ag<br>rv: tgt ggc tgt agg tgt cat cg
Gadd34	fw: ggc ggc tca gat tgt tca aa

	rv: ctg ccc aga cag caa gga aa
Xbp1u	fw: cag act atg tgc acc tct gc<br>rv: gtg tca gag tcc atg gga
Xbp1s	fw: tga gaa cca gga gtt aag aac acg<br>rv: cct gca cct gct gcg gac

II. Supplementary Results

Suppl. Table 2: Characteristics of subjects included for skeletal muscle quantitative polymerase chain reaction analyses.

**Parameter**	**Control**	**Insulin resistance**	**Sepsis**	***p*-value**
n [% male]	10 (30)	10 (90)	10 (70)	-
Age [years]	59±2	67±2	67±3	0.015[a,b]
BMI [kg/m^2]	24.4±0.9	27.9±0.6	28.3±1.4	0.027[b]
CRP [mg/l]	3.2±0.7[a]	8.7±2.1	192.9±31.7	<0.001[b,c]
IL-6 [pg/ml]	6.5±1.6	4.3±0.9	685.2±243.8	<0.001[b,c]
White blood cell count [x10^3/µl]	5.7±0.5	6.2±0.5	19.0±1.8	<0.001[b,c]
Platelet count [x10^3/µl]	232.5±24.0	214.7±24.5	406.8±44.8	<0.001[b,c]
HbA1$_c$ [%]	5.5±0.2	8.2±0.6	5.8±0.3	<0.001[a,c]
HbA$_{1c}$ [mmol/mol]	36.7±1.7	65.7±7.0	39.4±3.3	<0.001[a,c]
HOMA-IR [AU]	1.4±0.3	7.5±2.4	5.3±1.7	0.02
Fasting glucose [mmol/l]	7.3±0.5	10.8±0.9	7.6±1.1	<0.001[a,c]
Creatinine [µmol/l]	66.5±2.6	92.5±7.0	216.3±39.6	<0.001[a,b,c]
Albumin [g/l]	38.6±1.0	37.1±1.0	20.6±1.6	<0.001[b,c]
Bilirubin [µmol/l]	11.5±2.1	9.2±1.3	19.7±4.6	0.10
ALAT [µmol/l]	0.6±0.1	0.6±0.1	0.7±0.1	0.91
γGT [µmol/l]	0.8±0.1	2.6±1.1	3.4±0.9	0.18
Thromboplastin time [%]	108.6±4.2	105.2±4.7	76.9±2.2	<0.001[b,c]
Prior Laparotomy [%]	40	30	60	0.387

Malignancy [%]	70	80	70	0.84
Prior chemotherapy [%]	30	10	0	0.133
Prior radiotherapy [%]	20	0	0	0.117

Data are given as Mean ± SEM or absolute numbers. Superscript letters indicate significant differences ([a]control *vs.* IR, [b]control *vs.* sepsis, [c]IR *vs.* sepsis).

ALAT, alanine-aminotransferase; BMI, body mass index; CRP, C-reactive protein; GT, glutamyl-transferase; Hb, hemoglobin; HOMA-IR, Homeostatic Model of Insulin Resistance; IL, interleukin; different superscript letters indicate $p < 0.05$ between subgroups.

Suppl. Table 3: Indications for open abdominal surgery.

	Control	Insulin resistance	Sepsis
**Liver resection**			
benign liver disease	**4**	**2**	**-**
primary hepatic or biliary malignancy	**1**	**9**	**-**
secondary hepatic malignancy	**7**	**9**	**-**
**Gastrointestinal (GI) surgery**			
benign GI disease	**1**	**2**	**-**
GI malignancy	**3**	**5**	**-**
**Pancreatic surgery**			
Pancreatic carcinoma	**1**	**3**	**-**
**Other**			
Splenic metastasis	**0**	**1**	**-**
**Septic operations**			
Insufficiency of GI anastomosis	**-**	**-**	**2**
GI perforation	**-**	**-**	**3**
GI ischaemia	**-**	**-**	**1**
Insufficiency of pancreatic anastomosis	**-**	**-**	**3**
Necrotizing pancreatitis	**-**	**-**	**1**
Abscess	**-**	**-**	**3**

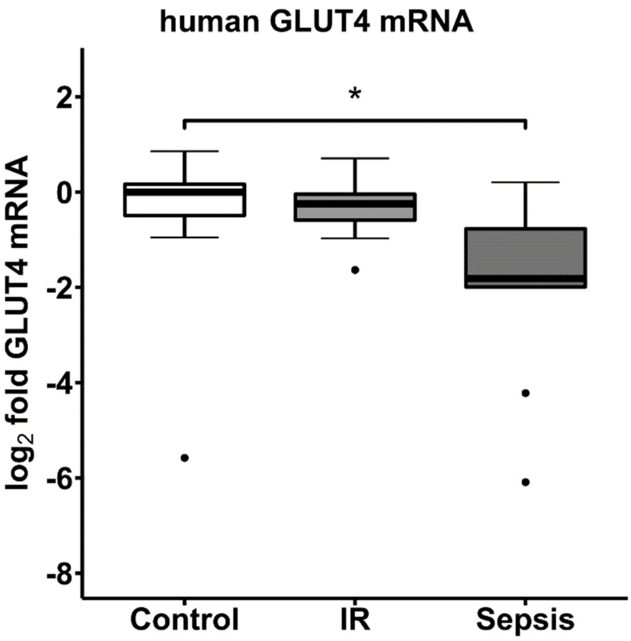

Suppl. Figure 1: GLUT4 mRNA in human skeletal muscle.

GLUT4 mRNA expression was significantly reduced in skeletal muscle of septic subjects compared to controls ($p<0.05$). Data of n = 10 subjects each of the matched cohort (see suppl. tab. 2).

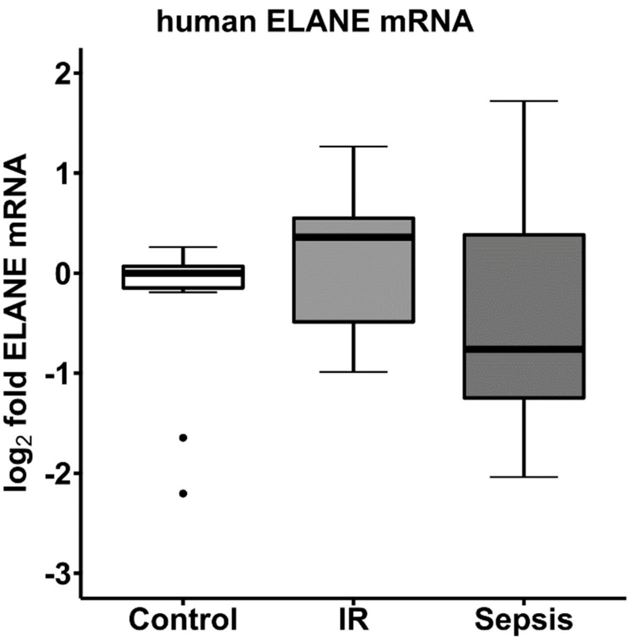

Suppl. Figure 2: ELANE mRNA in human skeletal muscle.

ELANE mRNA was not significantly different between groups (p>0.05).

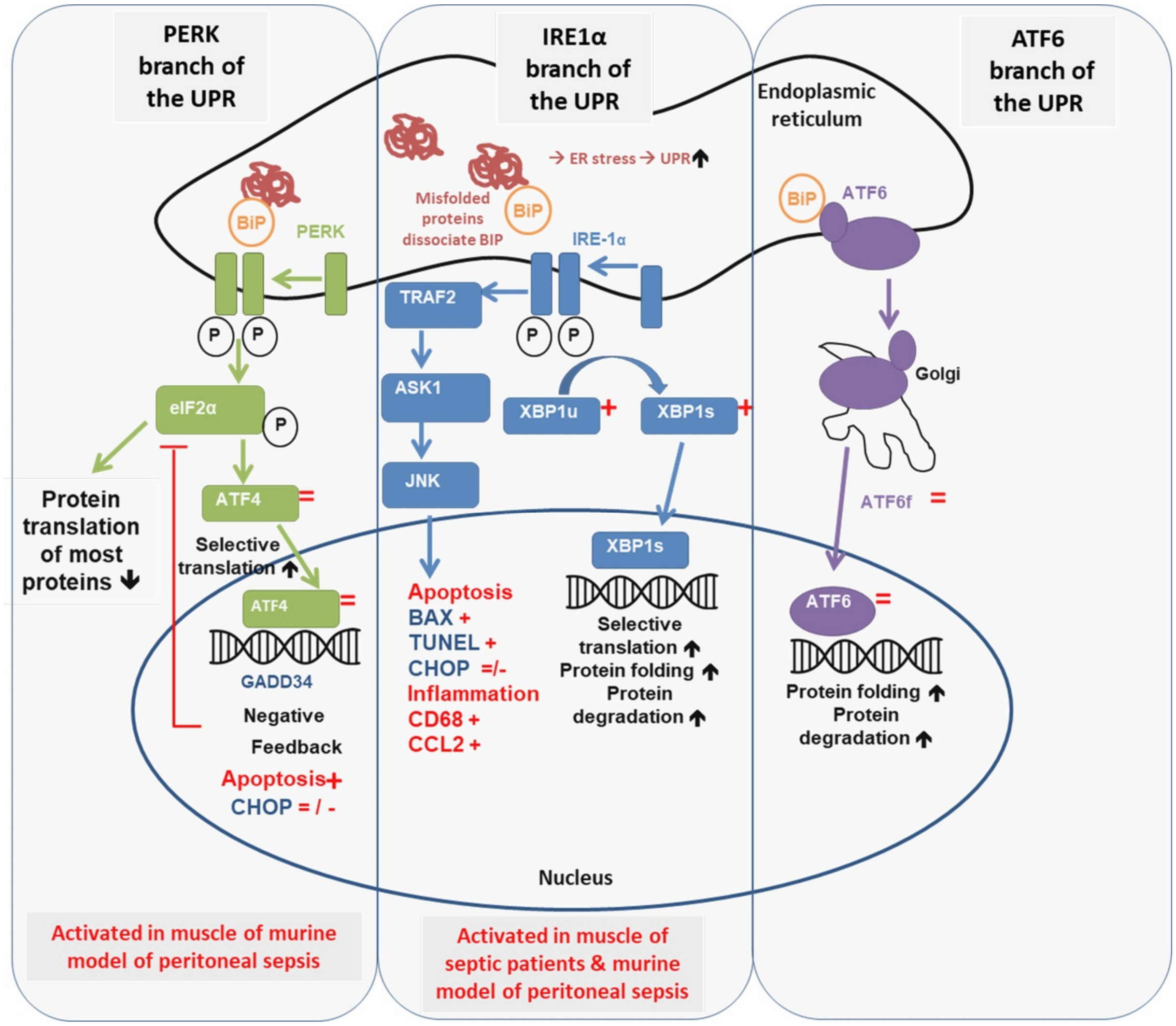

Suppl. Figure 3: UPR overview and effects of sepsis (red color) observed in the study.

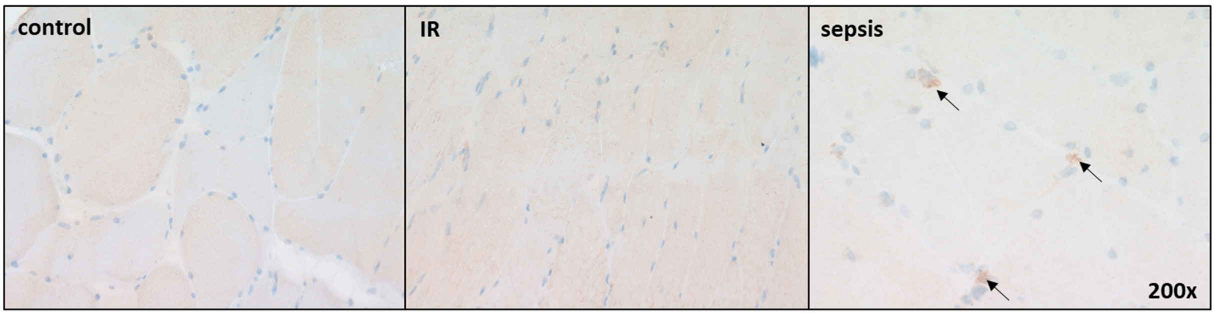

Suppl. Figure 4: XBP1 immunostaining in human skeletal muscle.

Staining of XBP1 enrichment in nuclei of human muscle tissue in a septic patient. Arrows indicate XBP1-positive nuclei (brownish coloring).

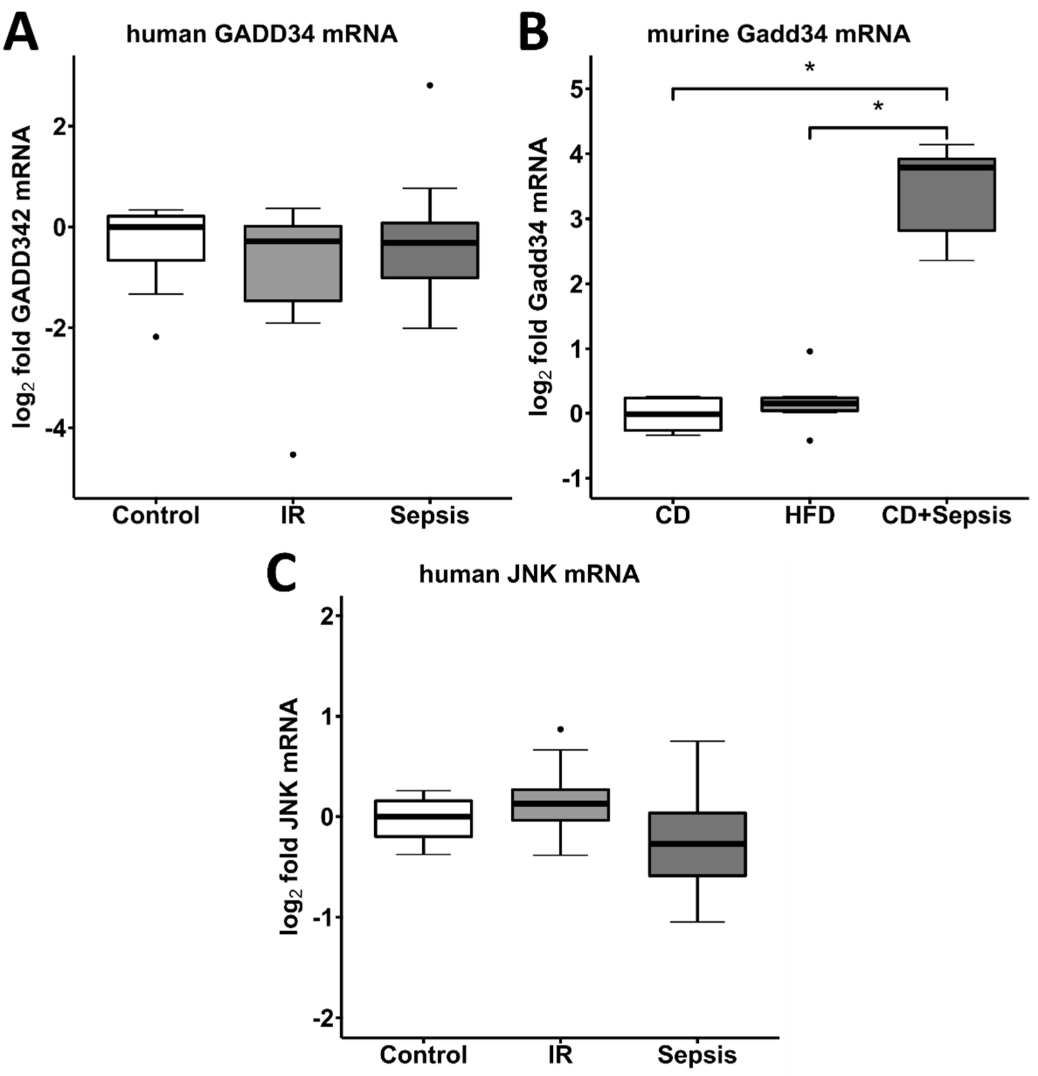

Suppl. Figure 5: GADD34, CASP12 and JNK mRNA expression in human and murine skeletal muscle.

A: Expression of GADD34 mRNA in skeletal muscle in patients with sepsis was not different compared to control and IR ($p>0.05$)

B: Expression of Gadd34 mRNA in skeletal muscle of a murine model of peritoneal sepsis was significantly increased compared to conditions of HFD ($p<0.001$) or control diet ($p<0.001$). No differences were observed in HFD vs. CD ($p>0.05$).

C: Skeletal muscle JNK mRNA levels in human skeletal muscle of patients suffering from peritoneal sepsis were comparable to control subjectss ($p>0.05$, respectively).

Plots represent data of n=5-6 for animals and n=10 for matched human data.

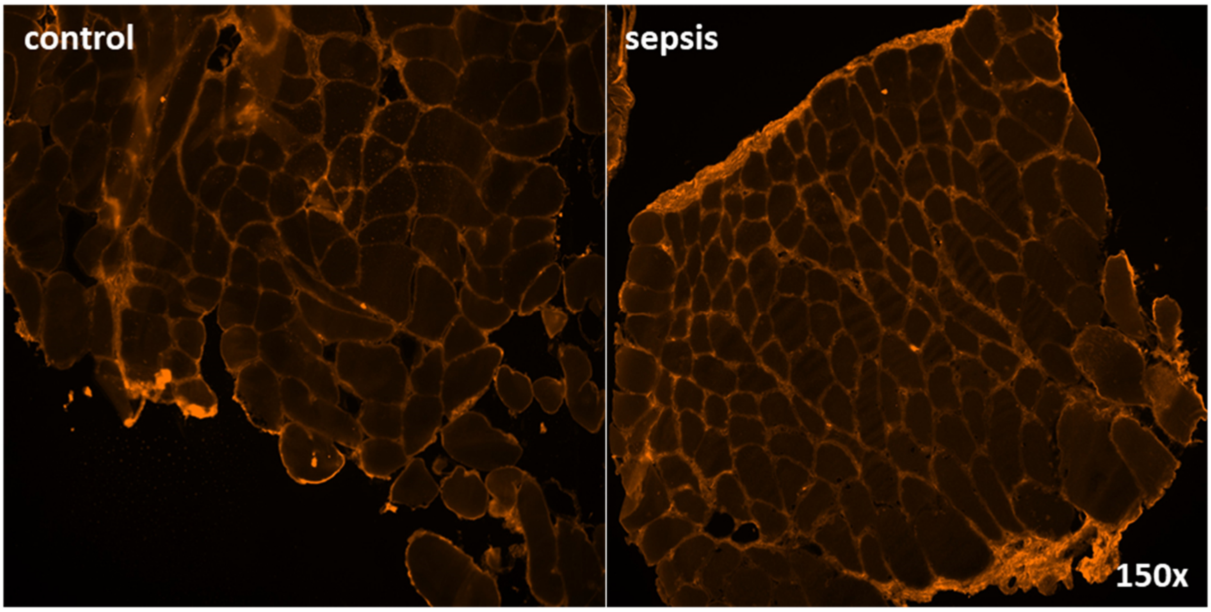

Suppl. Fig. 6: Immunostaining of CHOP in human skeletal muscle.

Representative staining of CHOP in control and septic skeletal muscle. No difference in semiquantitative analysis of staining intensity was observed between groups ($p>0.05$).

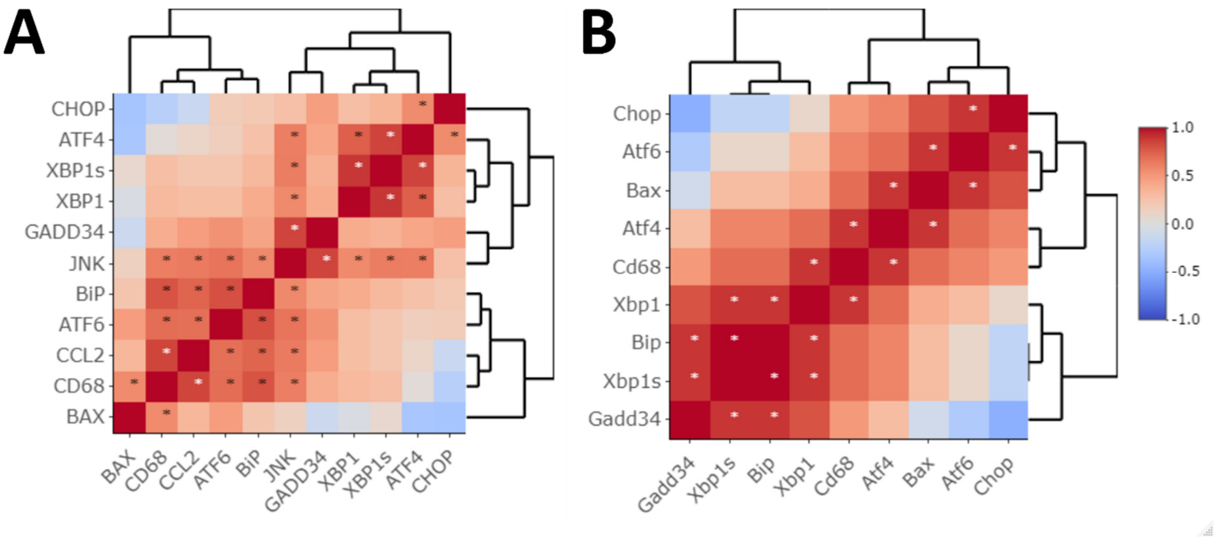

Suppl. Figure 7: Cluster correlation matrix analysis for (A) human and (B) murine sepsis. Colors indicate Spearman correlation coefficients for correlation of the log2 fold mRNA expression. Positive associations are shown in red, negative correlations in blue color with darker color indicating stronger associations. Dendrograms visualize complete-linkage hierarchical clusters. Color codes for Spearman correlation coefficient and asterisks indicate significant correlations (p>0.05).

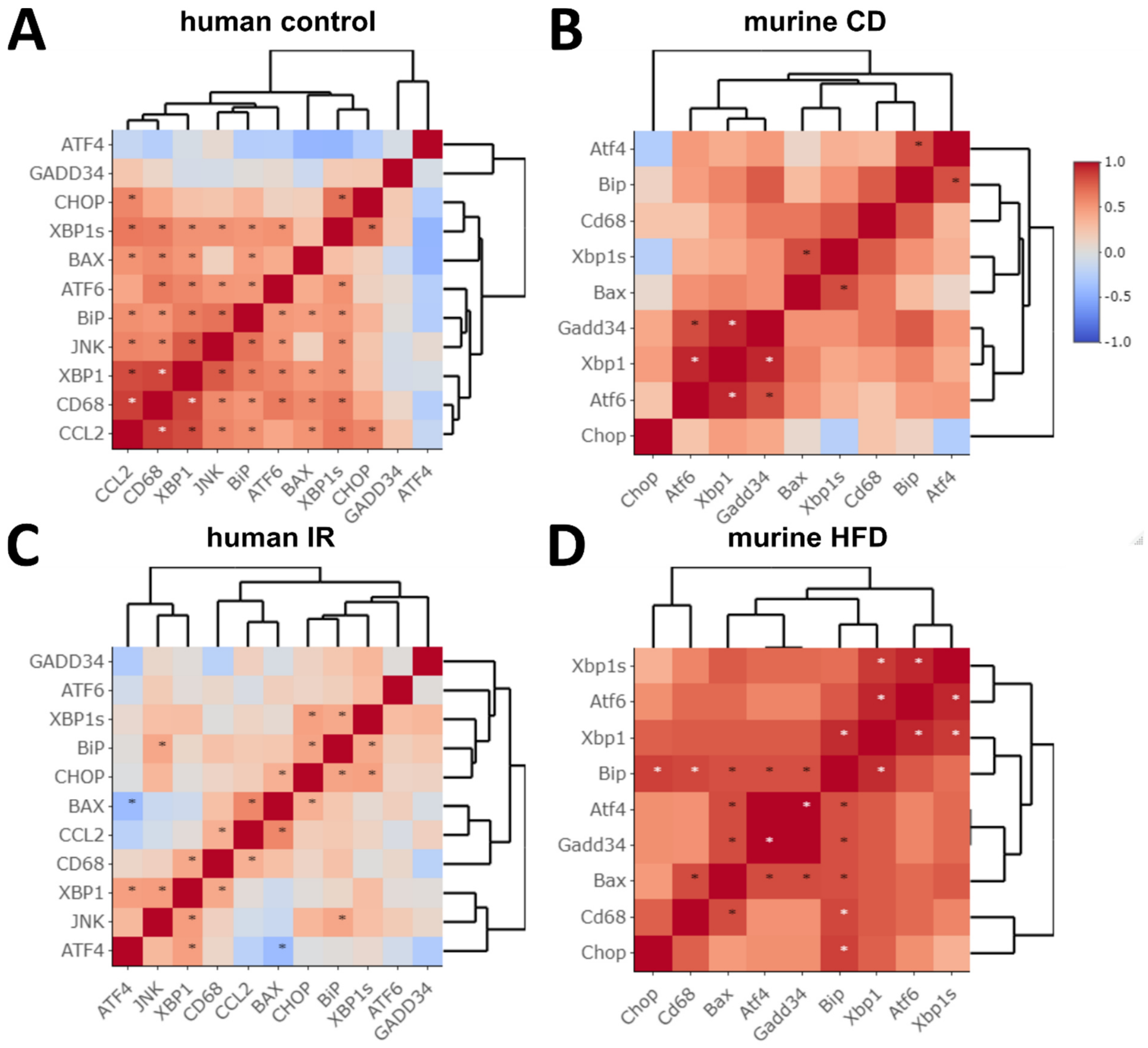

Suppl. Figure 8: Cluster correlation diagrams for human and murine control groups.

Color codes for Spearman correlation coefficient and asterisks indicate significant correlations (p>0.05).
